# Rhinovirus dynamics across different social structures

**DOI:** 10.1038/s44298-023-00008-y

**Published:** 2023-11-27

**Authors:** Martha M. Luka, James R. Otieno, Everlyn Kamau, John Mwita Morobe, Nickson Murunga, Irene Adema, Joyce Uchi Nyiro, Peter M. Macharia, Godfrey Bigogo, Nancy A. Otieno, Bryan O. Nyawanda, Maia A. Rabaa, Gideon O. Emukule, Clayton Onyango, Patrick K. Munywoki, Charles N. Agoti, D. James Nokes

**Affiliations:** 1 Epidemiology and Demography Department, KEMRI-Wellcome Trust Research Programme, Centre for Geographic Medicine Research – Coast, Kilifi, Kenya; 2 Department of Biochemistry and Biotechnology, Pwani University, Kilifi, Kenya; 3 Population & Health Impact Surveillance Group, KEMRI-Wellcome Trust Research Programme, Nairobi, Kenya; 4 Centre for Health Informatics, Computing, and Statistics, Lancaster Medical School, Lancaster University, Lancaster, UK; 5 Department of Public Health, Institute of Tropical Medicine, Antwerp, Belgium; 6 KEMRI-Centre for Global Health Research, Kisumu, Kenya; 7 Coronavirus and Other Respiratory Viruses Division (CORVD), National Center for Immunization and Respiratory Diseases (NCIRD), U.S. Centers of Disease Control and Prevention (CDC), Atlanta, GA USA; 8 U.S. Centers of Disease Control and Prevention (CDC), Nairobi, Kenya; 9 Department of Public Health, Pwani University, Kilifi, Kenya; 10 School of Life Sciences and Zeeman Institute for Systems Biology and Infectious Disease Epidemiology Research (SBIDER), University of Warwick, Coventry, UK; 11 Present Address: School of Biodiversity, One Health and Veterinary Medicine, University of Glasgow, Glasgow, G12 8QQ UK

**Keywords:** Epidemiology, Viral epidemiology

## Abstract

Rhinoviruses (RV), common human respiratory viruses, exhibit significant antigenic diversity, yet their dynamics across distinct social structures remain poorly understood. Our study delves into RV dynamics within Kenya by analysing VP4/2 sequences across four different social structures: households, a public primary school, outpatient clinics in the Kilifi Health and Demographics Surveillance System (HDSS), and countrywide hospital admissions and outpatients. The study revealed the greatest diversity of RV infections at the countrywide level (114 types), followed by the Kilifi HDSS (78 types), the school (47 types), and households (40 types), cumulatively representing >90% of all known RV types. Notably, RV diversity correlated directly with the size of the population under observation, and several RV type variants occasionally fuelled RV infection waves. Our findings highlight the critical role of social structures in shaping RV dynamics, information that can be leveraged to enhance public health strategies. Future research should incorporate whole-genome analysis to understand fine-scale evolution across various social structures.

## Introduction

Rhinoviruses (RV) are common respiratory pathogens transmitted via inhalation of contaminated aerosols or direct person-to-person contact^1^. They are positive-sense, single-stranded RNA viruses, with a genome ~7.2 kb long and a mutation rate ranging 10^−3^ to 10^−5^ mutations per nucleotide per genome replication event^2^. RV are classified into 169 types^3,4^, which are spread across three species: RV-A, RV-B, and RV-C. The types found in species A and B are proven to be antigenically unique from each other. However, for species C, this antigenic distinctiveness is yet to be confirmed^5^. RV-A and some RV-B utilize the intercellular adhesion molecule-1 (ICAM-1) as their receptor^1^, while RV-C uses a cadherin-related family member 3 (CDHR3) as their cellular receptor^2^. Rhinovirus prevalence in samples of individuals presenting with acute respiratory illness is estimated to be between 13–59% globally^6–10^, and 10–38.3% in Kenya^11–15^. Previously thought to cause only mild and self-resolving common-cold syndrome, RV are also an established cause of severe respiratory illnesses in both children and adults^8,10,16,17^. There is no approved RV antiviral, and vaccine development efforts have been hampered by the degree of antigenic diversity^3^.

Current epidemiological understanding of RV is restricted to the genetic diversity and transmission within individual settings such as hospitals, schools, or households^7,15,18–21^. RV infections occur year-round and are characterized by localized type-specific ‘mini-epidemics’^21,22^. Numerous types co-circulate within a setting, and their profiles change temporally^13,23^. Community social structures shape contact patterns^24^, thus shaping infectious disease transmission^25^. There is limited understanding of how RV circulation dynamics compare across different social structures and the degree to which local studies reflect the wider community at a national or global level. The design of effective non-pharmaceutical intervention strategies against RV can be improved by a detailed comprehension of its transmission dynamics across different social structures.

Tracking the spread of viral respiratory infections using nucleotide sequence data has become a useful tool to inform public health interventions on outbreak management. We aimed to improve understanding of RV dynamics across four social structures in Kenya of variable geographical coverage: (i) households in a single administrative location, (ii) a public primary school, (iii) outpatient clinics in a Health and Demographics and Surveillance System (HDSS), with (i)-(iii) all from rural coastal Kenya (Kilifi County) and (iv) hospital inpatients and outpatients across Kenya. Given the abundance of RV infections, understanding its dynamics across social structures will inform design and delivery of future interventions. Furthermore, RV dynamics might act as a proxy for other similarly transmitted but less frequent or possibly more severe respiratory viruses, such as influenza A, respiratory syncytial virus, and SARS-CoV-2.

## Materials and methods

### Studies and ethics

**Fig. 1:**
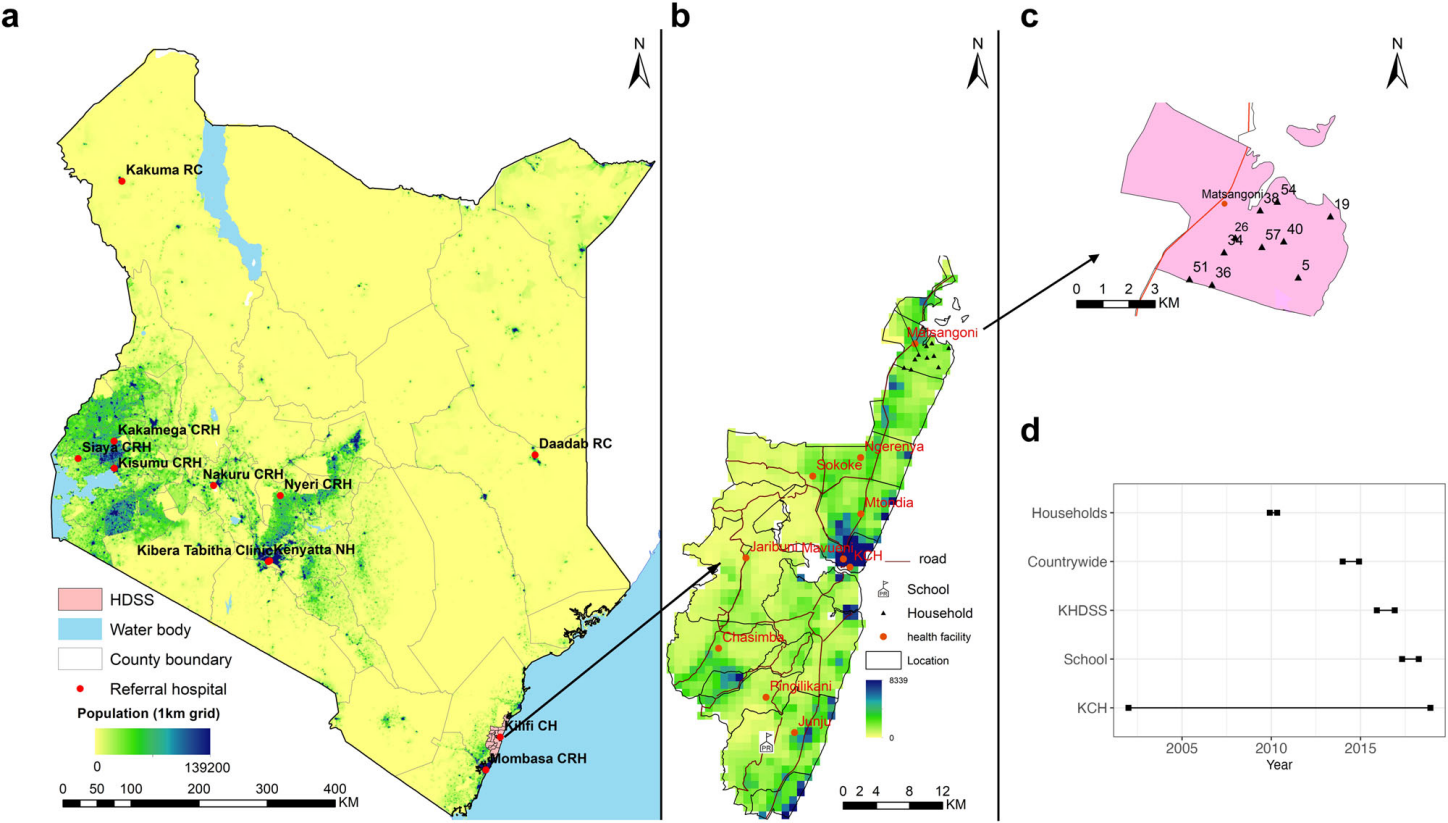
Geographical setting and time span of the studies included in the analysis. **a** A map of Kenya highlighting the countrywide study sites; **b** the Kilifi HDSS outpatient study sites, also showing the school location in Junju and the Kilifi County Hospital (KCH). **c** Households within Matsangoni location of the Kilifi Health and Demographics Surveillance System (HDSS). The map (**a**–**c**) is author created in ArcMap V.10.5 (ESRI, Redlands, California, USA). **d** Study periods of the included studies. Only contemporary KCH samples were included in the analysis i.e., KCH samples collected during the household, Kilifi HDSS and school studies. CRH County Referral Hospital, RC Refugee Camp, KCH Kilifi County Hospital.

We analysed sequence data from five studies across Kenya, cumulatively undertaken across four social structures (Fig. 1): (i) intensive household surveillance in a rural location in coastal Kenya^19^, (ii) surveillance of respiratory viruses within a school setting^21^, (iii) outpatient surveillance of acute respiratory illness (ARI) within the Kilifi HDSS^22^, and (iv) countrywide surveillance of severe acute respiratory illness (SARI) among inpatients and influenza-like illness (ILI) among outpatients via sentinel hospital reporting^26^. For comparison with the households, school and Kilifi HDSS studies’ datasets, we used contemporaneous data from (v) long-term surveillance of severe pneumonia among pediatric inpatients at the Kilifi County Hospital (KCH)^15,27–29^. The KCH data was collected during the same timeframe and was sourced from the same study population, i.e., rural coastal Kenya.

For each of these studies, informed written parental consent for persons under the age of 18 years or individual consent for adults was obtained before sample collection. All studies adhered to the principles of the Declaration of Helsinki. Ethical approvals were provided by the KEMRI-Scientific Ethics Review Unit, the University of Warwick Biomedical and Scientific Research Ethics Committee and the CDC Institutional Review Board: households (SSC #1651), school (KEMRI-SERU #3332 and BSREC #REGO_2016-1858); HDSS (KEMRI-SERU #3103 and BSREC #REGO-2015–6102); Countrywide (KEMRI-SERU #3044, CDC IRB #6806 and Project ID: 0900f3eb81e74404) and KCH (KEMRI-SERU #3443 and SSC #3178). The studies were approved to use pre-existent, pseudonymized specimens and data and had ethical approval for specimens to be tested to a broad range of respiratory pathogens.

The five studies above are distinct and independent of each other, and a summary of the individual studies and sampling for extensive RV analysis is described in Supplementary Table 1. Detailed information on study designs is described in previous publications^11,14,15,29–31^ and RV analyses have also been reported^13,19,21,22,26^. The geographic locations of the studies are depicted in Fig. 1.

### Data

The households study had 256 sequences (Dec 2009–May 2010), the school study had 256 sequences (May 2017–April 2018), the Kilifi HDSS study had 613 sequences (Dec 2015–Nov 2016), and the countrywide study had 803 sequences (Jan 2014–Dec 2014). Contemporaneous KCH sequences identified were: 73 sequences for the households study period, 66 sequences for the school study period and 81 sequences for the Kilifi HDSS study period.

We present previously unreported VP4/2 sequences (*n* = 225) of length ~420 bases from five households within the same location as (i) above, generated using the same laboratory protocol as the previous household data^19^ (protocol also described below). The new data were merged with prior household data for analysis. For phylogenetic comparison, we also included global VP4/2 sequences (*n* = 918) downloaded from the GenBank database. These were filtered to remove sequences from non-human samples, those shorter than 350 bases and sequences with missing metadata on date and location of sampling. Samples included in the analysis were from 35 countries across the world.

In total, 2373 Kenyan and 918 global VP4/2 sequences were analysed in this study. A breakdown of samples from each of the studies is provided in Supplementary Table 2.

### RV screening, sequencing, and type assignment

In all the studies above, RNA was extracted from nasopharyngeal swabs (NPS) and screened for respiratory viruses using a multiplex real-time reverse-transcription polymerase chain reaction (rRT-PCR)^27,32,33^. RV positivity was defined by a Ct-value of <35.0 for the school, Kilifi HDSS, KCH and countrywide studies, and <40.0 for the households study. The households study aimed to identify who infects whom and so it was important to be comprehensive in identifying infections, hence a lower threshold for viral titres was used for sequencing. RV-positive samples were amplified in the VP4/2 region and sequenced on an ABI 3130xl instrument (Applied Biosystems, USA). Forward and reverse complementary sequence reads were assembled into contigs using Sequencher version 5.4.6 (www.genecodes.com). RV type assignment was based on pairwise genetic distances as proposed (10.5% for HRV-A, 9.5% for HRV-B, and 10.5% for HRV-C)^34,35^ and phylogenetic clustering on Maximum Likelihood trees with prototype strains (www.picornaviridae.com/ensavirinae/enterovirus/prototypes/prototypes.htm)^13,19,21,22,26^.

### Comparison of RV-type spatial and temporal dynamics

Data analysis was performed using R version 4.2.1 (CRAN R Project). Categorical variables were summarized into counts and proportions. Kernel densities were used to infer the temporal patterns and define type-specific mini-epidemic waves across administrative locations. A type-specific mini-epidemic wave was defined as infections of the same type occurring in the same spatial and temporal frame, with no more than 14 days between two subsequent samples, as illustrated in Fig. 5b.

The Jaccard similarity index^36^ was used to evaluate the similarity of RV types identified in two contemporaneous studies within Kilifi, e.g., distinct types identified in the KCH study between Dec 2009–May 2010 were compared to those identified in the households. The Jaccard index ranges from 0 to 1 and gauges the diversity/similarity of sample sets, with higher values indicating higher similarity.

### Phylogenetic analysis

Bayesian phylogenetic analysis was done for the five most frequent RV types per social structure that were detected in more than one spatial frame therein. Type-specific global sequences, where present, were merged with the local sequences. Sequence alignments were prepared using the default algorithm in MAFFT v7.480^37^ and manually curated after alignment. The best-fitting nucleotide substitution and site heterogeneity model for each alignment were determined using ModelFinder^38^ in IQ-TREE v2.0.3^39^ and applied to BEAST v1.10.4^40^. We specified an uncorrelated lognormal relaxed molecular clock. Each model was run for at least 100 million Markov Chain Monte Carlo (MCMC) iterations ensuring Effective Sample Size (ESS) values > 200. Maximum clade credibility (MCC) trees were identified using TreeAnnotator v1.10.4 after removal of 10% burn-in and visualized using the R package *ggtree*^41^.

RV transmission between discrete locations (continent level, except for Kenyan sequences which were excluded from Africa) was inferred using the Bayesian Stochastic Search Variable Selection (BSSVS) under a symmetric diffusion model in BEAST and thereafter summarized using SpreaD3 v0.9.7.1^42^. Significant transmission links between locations were defined as those with a Bayes Factor (BF) value > 3. To obtain a composite RV transmission signal, we averaged the BF values of significant transmission pathways across all RV types in the analysis.

### Intra-type and (type-specific) inter-wave diversity

The genetic diversity of viruses belonging to the same type (mean pairwise genetic distances) was calculated for each social structure using MEGA X^43^.

We further investigated the genetic diversity across different mini-epidemic waves of the same type in the same spatial frame. Starting with the assertion that the basic unit of transmission is an epidemic wave comprising a variant of a single type that enters and spreads within a local population and fades out, then multiple waves/peaks of the same type in the same location are because of separate introductions and should be identifiable as genetically different. This way, observed patterns for a single type are composites of multiple individual introductions, each spreading independently within the local community.

We performed this analysis on purposively select types with multiple identifiable mini-epidemic waves in the Kilifi HDSS (A15, C1, C11) and countrywide studies (A22, A34, A49). We used a method described by Konishi et al.^44^ that directly applies principal component analysis (PCA) to a sequence alignment. First, the difference between any two samples was calculated using Euclidean distances. PCA then summarized the distance matrix to identify the principal components and record distances between each combination of samples. Finally, the highest-contributing principal components were subjected to k-means clustering. The optimal number of clusters was determined using the within-cluster sum of squares (wss) index.

### Definition of terms

We adopted a flexible definition of the term social structures as differing arrangement of institutions where people live or interact with each other. We defined a phylogenetic cluster as a group of sequences collected either from the same administrative location or a similar timeframe, supported by a branching posterior probability of >0.95. These are labelled K_1_-K_n_ (Fig. 3) and are independent from genetic clusters identified using machine learning (unsupervised learning using k-means clustering), subsequently labelled Cluster 1-Cluster n. Unless otherwise stated, the term (genetic) clusters refers to machine learning clusters.

## Results

### Overview

The Kenyan sequences were classified into 161 distinct RV types, of which 157 were known, and four types were unassigned, i.e., they did not meet the proposed threshold to any prototype strain. The countrywide study had the highest number of distinct types (*n* = 114), followed by the Kilifi HDSS (*n* = 78), the school (*n* = 47) and ultimately, the households (*n* = 40). Next, we compared RV types circulating within the different social structures to contemporaneous data from KCH. RV detections in KCH during the household study period (Dec 2009–May 2010) were classified into 37 types, of which 11 (29.7%) were also present in the household samples. For the school study period (May 2017–April 2018), KCH RV detections were classified into 40 types, of which 27 (67.5%) were shared across the two study populations. Finally, KCH RV detections during the HDSS study (Dec 2015–Nov 2016) were classified into 38 types, of which 31 (81.5%) were shared across the study populations. We used the Jaccard similarity index to compare types observed in KCH to those observed in the respective contemporaneous study. The highest similarity/ Jaccard index score was between the KCH and school study (*n* = 0.44), followed by KCH and the HDSS study (*n* = 0.37), and finally, the KCH and household study (*n* = 0.17).

### Temporal and spatial variation

**Fig. 2:**
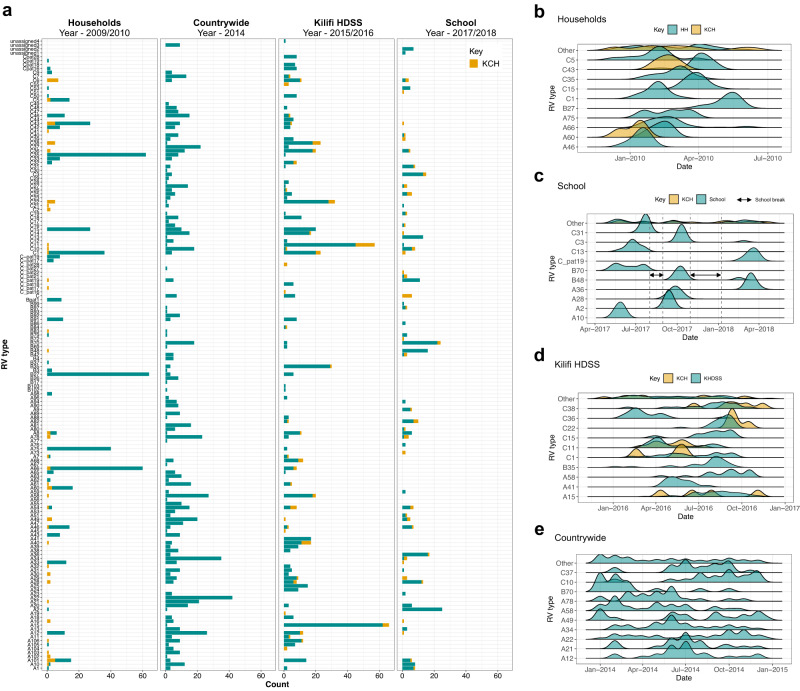
Summary of rhinovirus (RV) types observed. **a** Frequency counts of distinct RV types across the scales of observation. The studies are ordered by study year i.e., household (Dec 2009–May 2010), countrywide (Jan 2014–Dec 2014), Kilifi HDSS (Dec 2015–Nov 2016) and school (May 2017–April 2018). Contemporaneous KCH samples are coloured orange. **b**–**e** Temporal kernel density distributions of frequent RV types within the households, school, Kilifi HDSS, and countrywide study periods, respectively. Contemporaneous KCH samples are coloured orange. KHDSS Kilifi Health and Demographics Surveillance System.

Within each social structure, some types were notably more prevalent than others, and there was an evident turnover of the highly prevalent types in later years. In subsequent studies, previously frequent types disappeared or were detected in low frequencies (Fig. 2a). Starting with the earliest study, the households study (year 2009/10), the most prevalent types were B27 (*n* = 64, all detected in one household), C35 (*n* = 62, detected in 8 households) and A66 (*n* = 58, detected in 6 households). B27 was detected in very low frequencies in the countrywide study (year 2014) (*n* = 3, 0.4%) and the Kilifi HDSS study (year 2015/16) (*n* = 6, 0.9%) and none in the primary school (year 2017/18). Similarly, C35 was detected in low frequencies in the countrywide and Kilifi HDSS (*n* = 8 and *n* = 1, respectively) and none in the school study. On the other hand, A66 was not seen in the countrywide and school studies but was observed in the Kilifi HDSS study (*n* = 6). Interestingly, A58 was highly prevalent in both the countrywide (*n* = 27, 3.4%) and the Kilifi HDSS (*n* = 18, 2.9%) studies, Table 1. Of the 27 A58 samples identified in the countrywide study, only one was from Kilifi County, which was the catchment area for the Kilifi HDSS study. Overall, the high prevalence of types in one location was not associated with a high prevalence in other locations, probably the result of temporal changes in incidence.

**Table 1:** Frequencies of the ten most prevalent RV types per study and their respective frequencies in alternative studies.

Type	Households Year 2009/10 *n* = 482		Countrywide Year 2014 *n* = 803		Kilifi HDSS Year 2015/16 *n* = 613		School Year 2017/18 *n* = 256	
**Households**								
	Count (*n*)	Percent (%)	Count (*n*)	Percent (%)	Count (*n*)	Percent (%)	Count (*n*)	Percent (%)
B27	64	13.3	3	0.4	6	1.0	0	0.0
C35	62	12.9	8	1.0	1	0.2	0	0.0
A66	58	12.1	0	0.0	6	1.0	0	0.0
A75	40	8.3	0	0.0	2	0.3	0	0.0
C1	35	7.3	4	0.5	20	3.3	0	0.0
C15	27	5.6	10	1.3	20	3.3	0	0.0
C43	23	4.6	9	1.1	4	0.7	0	0.0
A46	13	2.7	3	0.4	2	0.3	6	2.3
A60	13	2.7	0	0.0	0	0.0	0	0.0
A33	12	2.5	7	0.9	0	0.0	0	0.0
C5	12	2.5	0	0.0	0	0.0	0	0.0
**Total**	**359**	**74.6**	**44**	**5.5**	**61**	**10.0**	**6**	**2.3**
**Countrywide**								
	Count (*n*)	Percent	Count (*n*)	Percent	Count (*n*)	Percent	Count (*n*)	Percent
A22	0	0.0	42	5.2	0	0.0	0	0.0
A34	0	0.0	35	4.4	0	0.0	1	0.4
A58	0	0.0	27	3.4	18	2.9	0	0.0
A12	11	2.3	26	3.2	10	1.6	0	0.0
A78	0	0.0	23	2.9	3	0.5	1	0.4
C37	0	0.0	22	2.7	3	0.5	0	0.0
A21	0	0.0	21	2.6	0	0.0	0	0.0
A49	0	0.0	20	2.5	0	0.0	3	1.2
C10	0	0.0	18	2.2	1	0.2	6	2.4
B70	0	0.0	18	2.2	2	0.3	22	8.6
**Total**	**11**	**2.3**	**252**	**31.4**	**37**	**6.0**	**33**	**12.9**
**Kilifi HDSS**								
A15	0	0.0	1	0.1	62	10.1	0	0.0
C11	0	0.0	1	0.1	45	7.3	1	0.4
B35	0	0.0	3	0.4	29	4.7	0	0.0
C22	0	0.0	1	0.1	28	4.6	0	0.0
C1	35	7.3	4	0.5	20	3.3	0	0.0
C15	27	5.6	10	1.2	20	3.3	0	0.0
A58	0	0.0	27	3.4	18	2.9	0	0.0
C36	0	0.0	12	1.5	18	2.9	4	1.6
C38	0	0.0	1	0.1	18	2.9	0	0.0
A41	0	0.0	0	0.0	17	2.8	0	0.0
**Total**	**62**	**12.9**	**60**	**7.5**	**275**	**44.9**	**5**	**2.0**
**School**								
	Count (*n*)	Percent	Count (*n*)	Percent	Count (*n*)	Percent	Count (*n*)	Percent
A2	1	0.2	1	0.1	0	0.0	25	9.9
B70	0	0.0	18	2.2	2	0.3	22	8.6
A36	0	0.0	3	0.4	0	0.0	16	6.3
B48	0	0.0	0	0.0	0	0.0	16	6.3
C13	0	0.0	1	0.1	0	0.0	13	5.1
C3	0	0.0	4	0.5	0	0.0	13	5.1
A28	0	0.0	5	0.6	6	1.0	12	4.7
C_pat19	0	0.0	5	0.6	0	0.0	11	4.3
A10	1	0.2	12	1.5	0	0.0	8	3.1
A1	1	0.2	3	0.4	2	0.3	7	2.7
A82	0	0.0	0	0.0	2	0.3	7	2.7
C31	0	0.0	3	0.4	1	0.2	7	2.7
unassigned2	0	0.0	0	0.0	0	0.0	7	2.7
**Total**	**3**	**0.6**	**55**	**6.8**	**13**	**2.1**	**164**	**64.1**

The studies are ordered by year.

We observed varying type-specific spatial distributions. Across the four social structures, the contribution of the ten most frequent types to total infections varied greatly. The total proportion of all sequences comprised by the ten most prevalent types in each social structure was: 74.6% in the household study, 64.1% in the school study, 44.9% in the HDSS study, and 31.4% in the countrywide study, Table 1.

We used kernel density distributions to demonstrate the spatial and temporal circulation and persistence of rhinovirus types within the different social structures (Fig. 2b–e). Frequent types in the households and school had uni-modal or bi-modal distributions. In contrast, RV types within the countrywide and Kilifi HDSS social structures showed multi-modal distributions stretched over extended periods. The sustained type-specific circulation at the HDSS and country levels was marked by mini-epidemics that were synchronised temporally and restricted geographically. For instance, in the HDSS study, C22 emerged and disappeared synchronously across the various administrative locations, while C38 was limited to the study locations in the northern region (Ngerenya, Sokoke, Mtondia and the KCH) (Supplementary Fig. 1A). At the countrywide level, some neighboring counties also exhibited potentially synchronous circulation of types, e.g., counties in Western Kenya showed similar temporal distributions of types A58 (Kisumu, Siaya and Kakamega), A21 (Kisumu and Siaya) and A78 (Kisumu and Kakamega) (Supplementary Fig. 1B). Although this distribution was characterized by numerous type-specific genetic clusters in co-circulation, sequences from the synchronous peaks were more closely related, suggesting inter-county spread. However, other RV types, e.g., A15 and C11, appeared to circulate randomly in the different locations with no defined temporal or spatial pattern.

### Local and global transmission dynamics

We performed phylogenetic analysis on 863 Kenyan and 918 global sequences representing 19 RV types (selected by identifying the five most frequent types per social structure), collected between August 1996 and April 2018. At a continent level, Europe had the highest number of sequences (*n* = 340), followed by Asia (*n* = 293) and Oceania (*n* = 205), while the rest of Africa (excluding Kenya), North and South America had less than 35 sequences each, Supplementary Table 3.

**Fig. 3:**
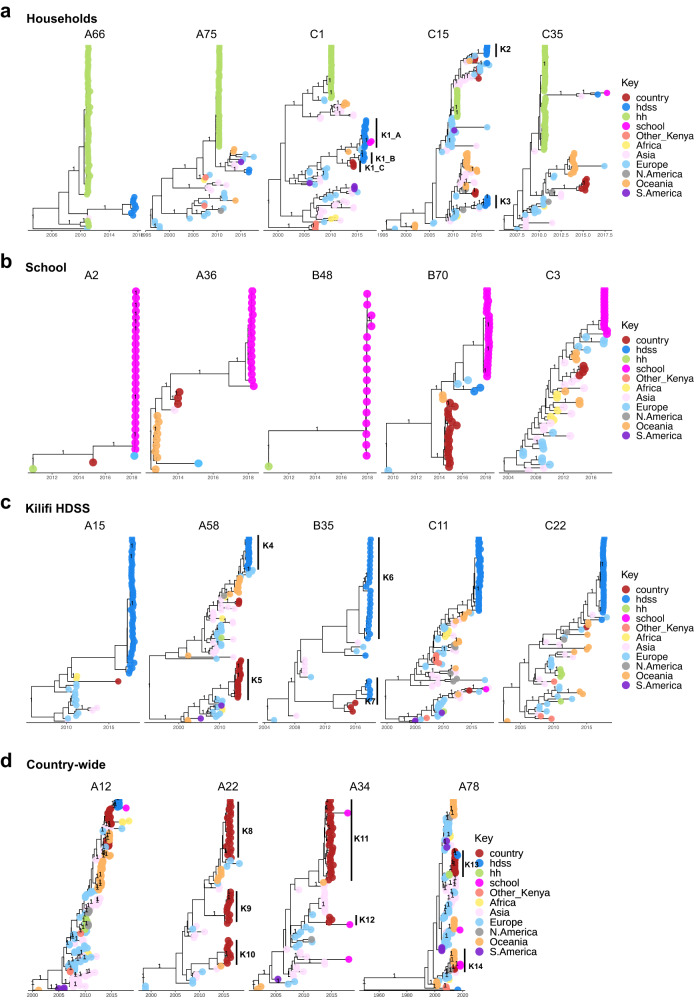
Type-specific time-inferred Maximum Clade Credibility (MCC) phylogenetic trees of frequent types observed in each social structure. **a** Highlights frequent types in the households study, **b** The school study, **c** The Kilifi HDSS, and **d** Countrywide settings. Across all panels, a total of 918 global sequences were included in the analysis. Tips are coloured by location and branching posterior probabilities greater than 0.95 are displayed. Phylogenetic clusters of interest are labelled K1 - Kn. The x-axis is scaled to the branch length in units of year. A58 was a common type for both the Kilifi HDSS and countrywide studies, and we therefore included one phylogenetic tree highlighting viruses from both studies. hh Households, hdss Health and Demographics Surveillance System.

In the type-specific phylogenies, the school and household viruses comprised single phylogenetic clusters on the global MCC tree, Fig. 3. In contrast, viruses from the Kilifi HDSS and countrywide studies occasionally fell into multiple phylogenetic clusters, e.g., the C15 and B35 viruses from the HDSS appeared as two phylogenetic clusters each (K2 and K3, K6 and K7, respectively), while A22, A34 and A74 viruses from the countrywide study fell into three (K8-K10), two (K11 and K12) and two (K13 and K14) phylogenetic clusters, respectively. Sequences from a given location or similar time frames were often found in more than one phylogenetic cluster. Temporal clustering of global and local sequences was only observed in A2, between viruses from the school study and Europe.

Locally, some RV types suggested in situ evolution and virus persistence. For instance, A36 viruses in the school (2017/18) shared recent ancestry with countrywide viruses from 2014. Also, while households C1 viruses clustered separately with those from the HDSS, some of the HDSS C1 viruses shared recent ancestry with those from the school (K1_A). Moreover, K1_B HDSS viruses shared recent ancestry with those from the countrywide study (K1_C). While this observation may be due to limited availability of contemporaneous data, we included all publicly available global data in the analysis to boost our confidence in the observations. In contrast, A58 viruses common in the countrywide and HDSS studies did not share the most recent ancestry, suggesting independent introductions, Fig. 3.

**Fig. 4:**
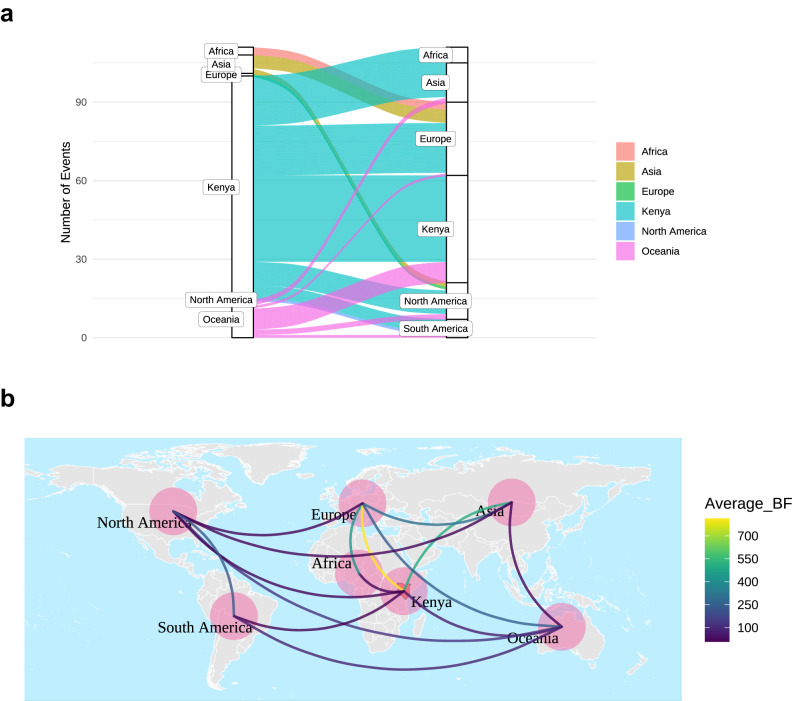
Rhinovirus transmission links. **a** Alluvium plots showing the estimated number and flow of RV transmission across the globe. ‘Africa’ refers to origins or destinations in African countries excluding Kenya. **b** A global map displaying average Bayes Factor (BF) values of significant transmission links across 19 select RV types. The color of the arc correlates to the average BF value.

We used Bayesian Stochastic Search Variable Selection under a symmetric diffusion model to infer potential virus transmission links. Most virus transmission links (*n* = 33) were potentially local transmission, i.e., within Kenya. The highest number of international transmission events were between Kenya - Europe (*n* = 19), Kenya - Asia (*n* = 13), and Kenya - Oceania (*n* = 8), Fig. 4a. Relative to sequence numbers, Kenya and Europe displayed 16 transmission links per 1000 sequences, Kenya and Asia had 11 transmission links per 1000 sequences and Kenya and Oceania had 7 transmission links per 1000 sequences. Using the mean Bayes Factor (BF) value, we identified the strongest transmission links as Kenya-Europe (BF value = 814), Kenya-Asia (BF value = 511) and Europe-rest of Africa (BF value = 436), Fig. 4b.

### Intra-type and (type-specific) inter-wave diversity

**Fig. 5:**
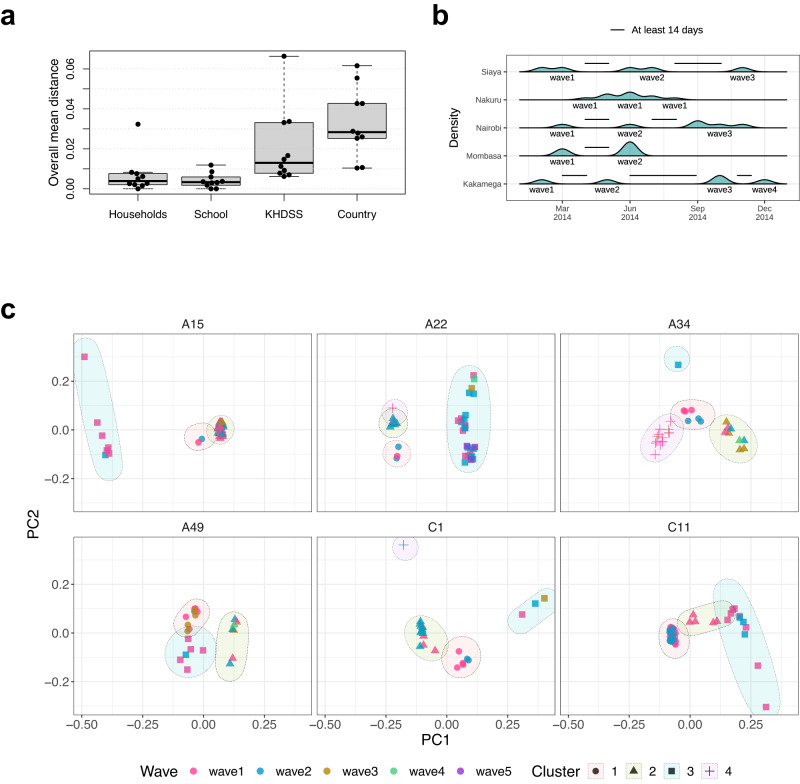
Genetic diversity of rhinovirus infections. **a** Distribution of the overall mean distance of frequent types in different social structures. **b** An illustration of type-specific mini-epidemic wave definition. Consecutive samples of the same type in the same location/county collected more than 14 days apart were classified into different epidemic waves. **c** K-means clustering of principal components (PC) performed on six select RV types. Each dot represents a sample. Samples are coloured by their respective epidemic wave. The respective genetic cluster is highlighted by a shaded ellipsis.

We compared the genetic diversity of the ten most frequent RV types across the four social structures. The intra-type genetic diversity was lowest at the school level (range 0–1.2%, median = 0.3%), followed by the households (range 0–3.2%, median = 0.4%), the Kilifi HDSS (range 0.6–6.6%, median = 1.3%), and highest at the countrywide level (range 1.0–6.2%, median = 2.8%), Fig. 5a. This diversity correlated with the size of geographical space represented by the respective study, i.e., the school represented a single point within a location, households were distributed within a single location, the Kilifi HDSS study sites provided a representation of an area of 891 km^2^ and the countrywide study was a broad representation of Kenya.

We performed PCA and k-means clustering for six select types to identify genetic clusters of circulating infections within given geographic locations. All types included in the analysis displayed genetic diversity, which was refined as three clusters for A15, A49 and C11, and four clusters for A22, A34 and C1, Fig. 5 c.

We zoomed in to the administrative location for the HDSS or county levels for the countrywide study and made three noteworthy observations (Supplementary Fig. 2). First, there was an apparent geographic clustering of infections, suggesting introduction of a single variant of a type and rapid spread within the community. For instance, Cluster 1 of A15 was limited to Mtondia, Ngerenya, and Sokoke, which are neighbouring locations of the HDSS. Similarly, Cluster 3 of the same type was only detected in KCH and Mtondia. At the countrywide level, Cluster 3 of A34 was limited to Nakuru and Kakamega, Cluster 2 of A22 to Kakuma, and while Cluster 1 of A49 was predominantly in the coastal region (Mombasa and Kilifi), three samples were also detected in Nairobi. Secondly, some clusters were more common and widely distributed, e.g., Cluster 2 of A15 was detected in 8 of 9 administrative locations of the HDSS, as well as the KCH. In addition, Cluster 3 of A22 and Cluster 2 of A34 were detected in seven and five counties, respectively. Finally, other clusters were detected in distant locations, e.g., Cluster 1 of A22 was observed in Kilifi, Nairobi and Siaya and Cluster 3 of C1 was detected in Matsangoni, Mtondia and Ngerenya, suggesting transmission due to human travel/migration.

We further investigated the genetic diversity of a single type to test the hypothesis that multiple epidemic waves for a given type in a location are because of separate introductions and should be identifiable as genetically different. At the location or county levels, 56/84 (66.7%) mini-epidemic waves were caused by a single genetic cluster, 24/84 (28.6%) waves were caused by two clusters, and 4/84 (4.8%) were caused by more than two clusters. For counties/locations that experienced more than one type-specific wave, a total of 15 mini-epidemic waves were either solely or jointly (alongside a new genetic cluster) caused by genetic clusters detected in the previous wave(s). Similarly, an equal number of waves (*n* = 15) were composed of new genetic clusters of the same type, not previously detected in the region, Supplementary Fig. 2.

Some RV types displayed minimal inter-cluster variance in given locations/counties. In particular, A34 infections in Nakuru were classified into three clusters, but these were very closely located along the PCA coordinate space, indicating minimal variance between the clusters. Different clusters of C11 infections from the KCH and Matsangoni also displayed minimal variance along the PCA coordinate space, Supplementary Fig. 2. On the other hand, A34 and C11 epidemic curves in Nakuru and KCH and Matsangoni, respectively, suggested propagated (progressive source) epidemics, Supplementary Fig. 1. The minimal inter-cluster variance, alongside the propagated epidemic curves, suggests that the virus may have diversified in situ within the community, creating a continuous source of genetically diverse infections.

## Discussion

We investigated rhinovirus patterns across four social structures of variable geographical coverage in Kenya to better understand its transmission and circulation dynamics. We observed a remarkable degree of genetic diversity within Kenya, documenting the occurrence of >90% of all known global RV types and four potentially new types. The country-level demonstrated the highest diversity (marked by the number of distinct types), followed by the Kilifi HDSS, school and household levels. Type-specific infections were also more diverse in the HDSS and countrywide studies compared to households and the school. As the Kilifi HDSS and countrywide levels encompass larger geographical areas, it is anticipated that they would experience more introductions of the virus. The frequency of these introductions may also be influenced by factors such as population density and the movement of people into and out of the specified geographical regions.

The prevalence of certain types in one location did not necessarily correlate with similar prevalence levels in other locations. Instead, there was a turnover of the most prevalent types, which was likely influenced by temporal type dynamics. The disappearance of types after predominance at broad geographical scales was consistent with the development of long-term type-specific immunity^45,46^. A58 was identified as a frequent type in both the countrywide study (year 2014) and the subsequent HDSS study (year 2015/16), the recurring A58 viruses belonged to a different phylogenetic cluster. This could be stochastic or because of a fitness advantage of the second A58 cluster over the first cluster. We propose the analysis of whole genomes to explore possible mutations in the antigenic viral proteins 1–3 that may contribute to increased rhinovirus fitness/transmissibility.

Frequent types in households and the school had either uni/bi-modal distributions in contrast to the countrywide and HDSS. The shorter epidemics in the school and household settings can be explained by the high contact patterns characterizing these settings^24,47^ facilitating faster spread of infections. The school and household studies also represented smaller geographical spaces (a single administrative location within the HDSS), and it is therefore anticipated that the circulating virus took a shorter time to exhaust susceptible individuals. The multi-modal distributions observed for larger geographical spaces were resultant of multiple epidemics varying temporally and spatially at smaller geographical frames therein, i.e., administrative locations in the HDSS and counties in the countrywide study. Larger geographical spaces had a higher diversity of type-specific RV infections. Our analysis showed that this was a factor of multiple concurrent introductions or subsequent introductions of genetically diverse type-specific viruses or potential in situ evolution of circulating viruses.

Interestingly, no contemporaneous pair of studies in Kilifi County detected an identical set of circulating RV types, as evidenced by the Jaccard index. This highlights the complexity of our communities and the role of social structures in shaping infectious disease dynamics, indicating that it requires multiple social structures to understand community transmission dynamics better. The highest similarity in detected types was observed between the KCH and school setting, followed by KCH and HDSS and, ultimately, KCH and households. One possible explanation for the similarity between the KCH and school settings is the similar age groups of the study participants: the sampled students were 3–19 years old (median 7 years), which resembles the pediatric cohort sampled at KCH (<60 months), suggesting a similar immune profile shaped by age.

Some geographically close locations, e.g., counties in Western Kenya, showed similar epidemic curves, indicating inter-county mixing and transmission amongst geographically co-located counties. Besides, infections from neighboring geographical regions occasionally fell into a single genetic cluster, suggesting a single introduction and rapid spread within the community. While contemporaneous global data were sparse, phylogenetic analysis showed that observed local RV infections were a result of both local transmission and virus importations. Perhaps due to limited contemporaneous global data, temporal clustering of global and local sequences was only observed between viruses from the school study and Europe (RV type A2). Transmission links across different continents suggest a potential role of human movement in influencing rhinovirus infections. We acknowledge that tree topologies are only a representation of a complex set of models and the viral sequence data and may not always accurately capture reality. Nevertheless, similar to our analysis, Kenya-Europe and Kenya-Asia were the most common transmission pathways previously noted by a study focusing on respiratory syncytial virus^48^. Europe is the leading source of tourists to Kenya^49^, and the recent increasing Chinese economic interest in Africa^50^ has resulted in increased human traffic between China and Africa, including Kenya.

This study also tested the hypothesis that multiple waves of a given type in the same location should be differentiable as genetically different. We made three observations. (i) The majority of type-specific waves at an administrative location or county level were caused by a single genetic variant of an RV type, suggesting a single introduction into the local population. (ii) We found evidence that subsequent waves of the same RV type are occasionally composed of new genetic variants but failed to reject the null hypothesis that type-specific waves deplete the susceptible population in a location. This may be due to slow sustained transmission that was not detected under our study design, or an influx of susceptible individuals from outside of the location. (iii) New genetic variants in the population were potentially a result of new introductions or in situ evolution; whether the new variants hold a fitness advantage is yet to be confirmed.

This analysis had some limitations. First, discrepancies in the individual study designs and differences in study periods across the different social structures made comparisons difficult. Second, the VP4/2 region is relatively short, which may result in a high statistical uncertainty when inferring transmission, as previously demonstrated^51^. Third, our approach to investigating global transmission dynamics of frequent RV types in Kenya was centered on publicly available sequence data. Although we included all available data for the analysed RV types, regions without sequence coverage were omitted from the global picture.

This study refines our understanding of how RV molecular diversity compares across different social structures, reinforcing the role of social structures in shaping infectious disease dynamics. We demonstrate that at large geographical spaces (county and country), rhinovirus is endemic, and infections are caused by highly diverse viruses. In addition, rhinovirus epidemics or waves are driven by multiple variants of RV types. This improved understanding can act as an indicator for comprehending the dynamics of other less frequent or potentially more severe respiratory viruses. Future studies should include whole-genome analysis to refine understanding on RV transmission and evolutionary dynamics at the various social structure levels.

### Supplementary information

Supplementary information

## Data Availability

Sequence data analysed in this article are available in GenBank under accession numbers: KX831136 - KX831389 and OL853844 - OL854069 (households), MT177659–MT177911 (school), MH459421-MH460237 (Kilifi HDSS), MZ129390 - MZ130096 (countrywide), KY006195 - KY006465 and MW622248 - MW623046 (KCH). Additional data and analysis scripts for this manuscript are available at the VEC Harvard Dataverse^52^.
