# Supplementary material for: Rhinovirus dynamics across different social structures
Source: Npj Viruses. 2023 Nov 27;1:6. doi: 10.1038/s44298-023-00008-y (PMC11041716; doi:10.1038/s44298-023-00008-y)
Supplement: Supplementary file 1 — Supplementary information [file 44298_2023_8_MOESM1_ESM.docx]

**Appendix**

**Supplementary Table 1: A summary of studies included in this analysis.**

| **Study** | **Study period** | **Location** | **Primary objective of study** | **Study participants** | **Detailed RV analysis using VP4/2 sequencing** | **Comments** |
| --- | --- | --- | --- | --- | --- | --- |
| The households study ^1^ | Dec 2009 - May 2010 | Households within Matsangoni location of the KHDSS. The location was chosen because of its convenient road accessibility and pre-existing community network, which enhanced community access and involvement. | To identify the source of infant respiratory syncytial virus (RSV) infections. | All ages regardless of respiratory symptoms. 47 households of various sizes (range, 4–37 members) were surveilled. All recruited household members were sampled twice weekly. | All RV positives from five households initially selected to represent the breadth of household sizes in the larger cohort ^2^. An additional five households were later selected using same criteria to increase study power. There were no significant differences between the sequenced subset against the non-sequenced groups in demographic or clinical characteristics. | Initial five households and their sizes were: HH19 (n=14), HH34 (n=7), HH40 (n=5), HH5 (n=37) and HH51 (n=15).  Additional five households were: HH26 (n=5), HH36 (n=16), HH38 (n=23), HH54 (n=7) and HH57 (n=16). |
| The school surveillance ^3^ | May 2017 – April 2018 | A public primary school within Junju location of the KHDSS. Junju was for its strong ties between residents, the local health facility, and research institute, KWTRP. | To study the  types and frequency of  respiratory viruses affecting school children. | All ages of pupils and teaching staff self-reporting with respiratory symptoms. Overall, 469 students aged 2-19 years were followed up over the study period (excluding school holidays). A maximum 8 samples per grade per week from the lower primary (5 grades) and 4 samples per grade per week from the upper primary (7 grades) were collected weekly. If symptomatic individuals per grade exceeded the set cap, randomization determined which individuals were sampled. | All RV positives were selected for sequencing ^4^ |  |
| The KHDSS outpatient study ^5^ | Dec 2015 - Nov 2016 | Nine dispensaries representing the KHDSS, covering variation in population density. | To understand community respiratory virus dynamics in outpatients within the KHDSS. | All ages presenting to health centers with acute respiratory infection. A weekly sample size of 15 samples per health center was set using previous outpatient data and past inpatient surveillance experience for respiratory viruses at KCH. | All RV positives were selected for sequencing ^6^. | Nine dispensaries were: Chasimba, Jaribuni, Mavueni, Mtondia, Ngerenya, Sokoke, Junju, Matsangoni and Pingilikani. |
| The countrywide study ^7^ | Jan 2014 - Dec 2014 | Twelve health centers across Kenya: ten referral hospitals (for inpatients) and two outpatient clinics. | To monitor severe acute respiratory syndrome across all age groups in Kenya. | All ages presenting to health centers with severe acute respiratory illness for inpatients or influenza-like illness for outpatients. | All RV positives were selected for sequencing ^8^. | Surveillance sites were:  Dadaab refugee camp, Kakuma refugee camp, Kilifi County Hospital (Kilifi), Mombasa County Hospital (Mombasa), Nyeri County Hospital (Nyeri), Nakuru County Hospital (Nakuru), Siaya County Hospital (Siaya), Kakamega County Hospital (Kakamega), Lwak Mission Hospital (Kisumu), Kibera Tabitha Clinic (Nairobi) and Kenyatta National Hospital (Nairobi). |
| KCH paediatric surveillance  ^9–12^ | 2002 to 2018 | KCH, which serves as the referral hospital for Kilifi County. | Surveillance of aetiology of severe pneumonia among paediatrics admissions | Children less than five years old admitted to the paediatric ward with symptoms of syndromic severe or very severe pneumonia. Samples were collected as soon as possible following hospital admission. | All RV positives were selected for sequencing ^13^. |  |
| **Abbreviations**:  RV - rhinovirus  KHDSS – Kilifi Health and Demographic Surveillance System  KCH – Kilifi County Hospital  KWTRP – KEMRI-Wellcome Trust Research Programme | | | | | | |

**Supplementary Table 2: VP4/2 sequences analysed in this study.**

| **Time period** | **Study** | **Number of sequences** | **Contemporaneous sequences from KCH** | **Total samples** |
| --- | --- | --- | --- | --- |
| Dec 2009 - May 2010 | Households study | 481 (of which 225 are newly generated) | 73 | 554 |
| May 2017 – April 2018 | School study | 256 | 66 | 322 |
| Dec 2015 - Nov 2016 | KHDSS study | 613 | 81 | 694 |
| Jan 2014 - Dec 2014 | Countrywide study | 803 | N/A | 803 |
| Aug 1996 - April 2018 | Global sequences | 918 | N/A | 918 |
| **Total** | | | | **3291** |
| Abbreviations:  KHDSS – Kilifi Health and Demographic Surveillance System  HH - household  KCH – Kilifi County Hospital  N/A - Not Applicable | | | | |

**Supplementary Table 3. Summary counts of sequences per RV type included in the phylogenetic analysis.**

| **Type** | **Kenya** | **(Rest of) Africa** | **Asia** | **Europe** | **North America** | **South America** | **Oceania** | **Total** |
| --- | --- | --- | --- | --- | --- | --- | --- | --- |
| **A12** | 52 | 6 | 67 | 60 | 7 | 3 | 55 | 250 |
| **A15** | 67 | 2 | 3 | 12 | 0 | 0 | 0 | 84 |
| **A2** | 27 | 0 | 0 | 1 | 0 | 0 | 0 | 28 |
| **A22** | 42 | 0 | 9 | 10 | 0 | 0 | 6 | 67 |
| **A34** | 38 | 0 | 23 | 11 | 1 | 1 | 3 | 77 |
| **A36** | 20 | 0 | 1 | 1 | 0 | 0 | 9 | 31 |
| **A58** | 48 | 4 | 20 | 26 | 1 | 2 | 14 | 115 |
| **A66** | 69 | 0 | 1 | 1 | 0 | 0 | 0 | 71 |
| **A75** | 44 | 1 | 6 | 16 | 1 | 1 | 3 | 72 |
| **A78** | 35 | 5 | 39 | 55 | 2 | 6 | 43 | 185 |
| **B35** | 33 | 0 | 6 | 4 | 0 | 0 | 0 | 43 |
| **B48** | 17 | 0 | 0 | 0 | 0 | 0 | 0 | 17 |
| **B70** | 44 | 0 | 0 | 3 | 0 | 0 | 2 | 49 |
| **C1** | 70 | 2 | 25 | 19 | 0 | 4 | 4 | 124 |
| **C11** | 68 | 5 | 22 | 27 | 6 | 3 | 11 | 142 |
| **C15** | 57 | 2 | 36 | 52 | 4 | 1 | 27 | 179 |
| **C22** | 41 | 0 | 8 | 15 | 2 | 0 | 9 | 75 |
| **C3** | 19 | 5 | 14 | 14 | 0 | 0 | 6 | 58 |
| **C35** | 72 | 1 | 13 | 13 | 2 | 0 | 13 | 114 |
| **Total** | **863** | **33** | **293** | **340** | **26** | **21** | **205** | **1781** |

### Supplementary Materials:

**Figures**

**
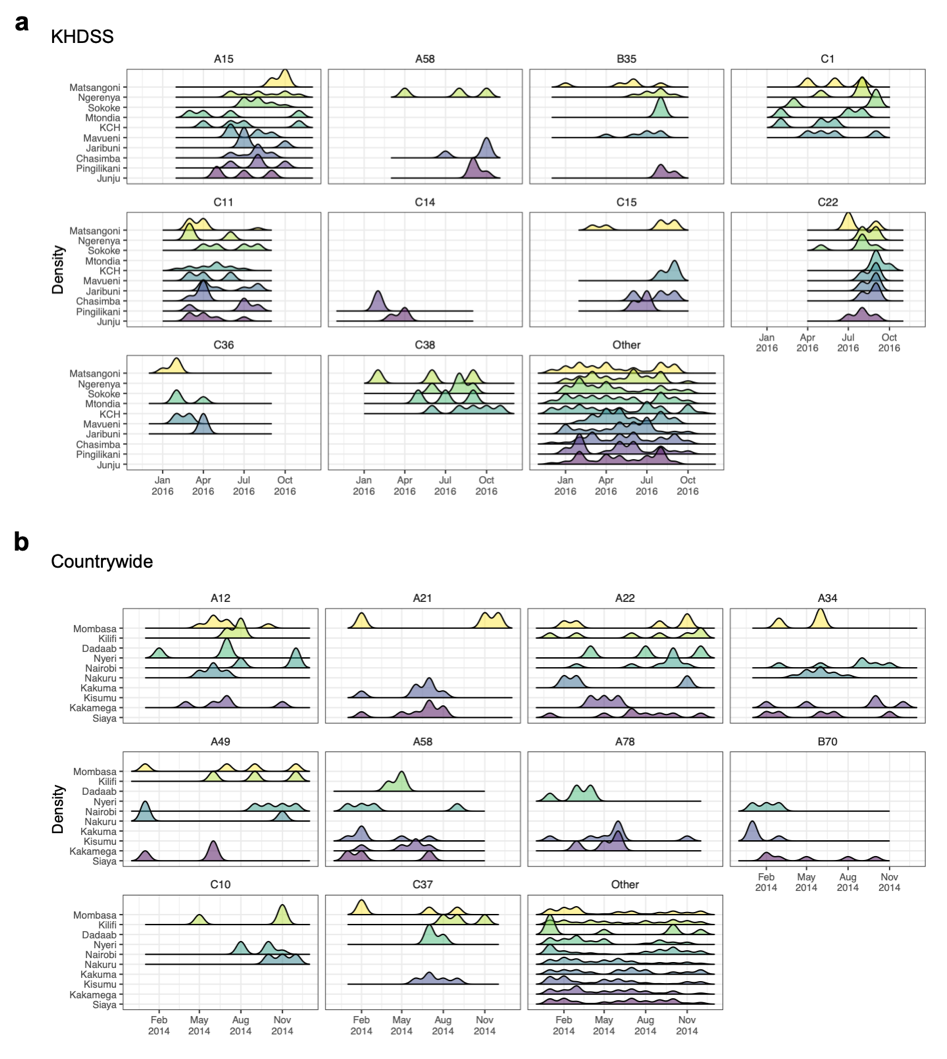
**

**Supplementary Figure 1.** Kernel distributions of rhinovirus types within (A) the Kilifi HDSS and (B) the countrywide studies. The ten most frequent types are displayed individually while less frequent types are summarised as “Other”.

Abbreviations: KHDSS – Kilifi Health and Demographic Surveillance System


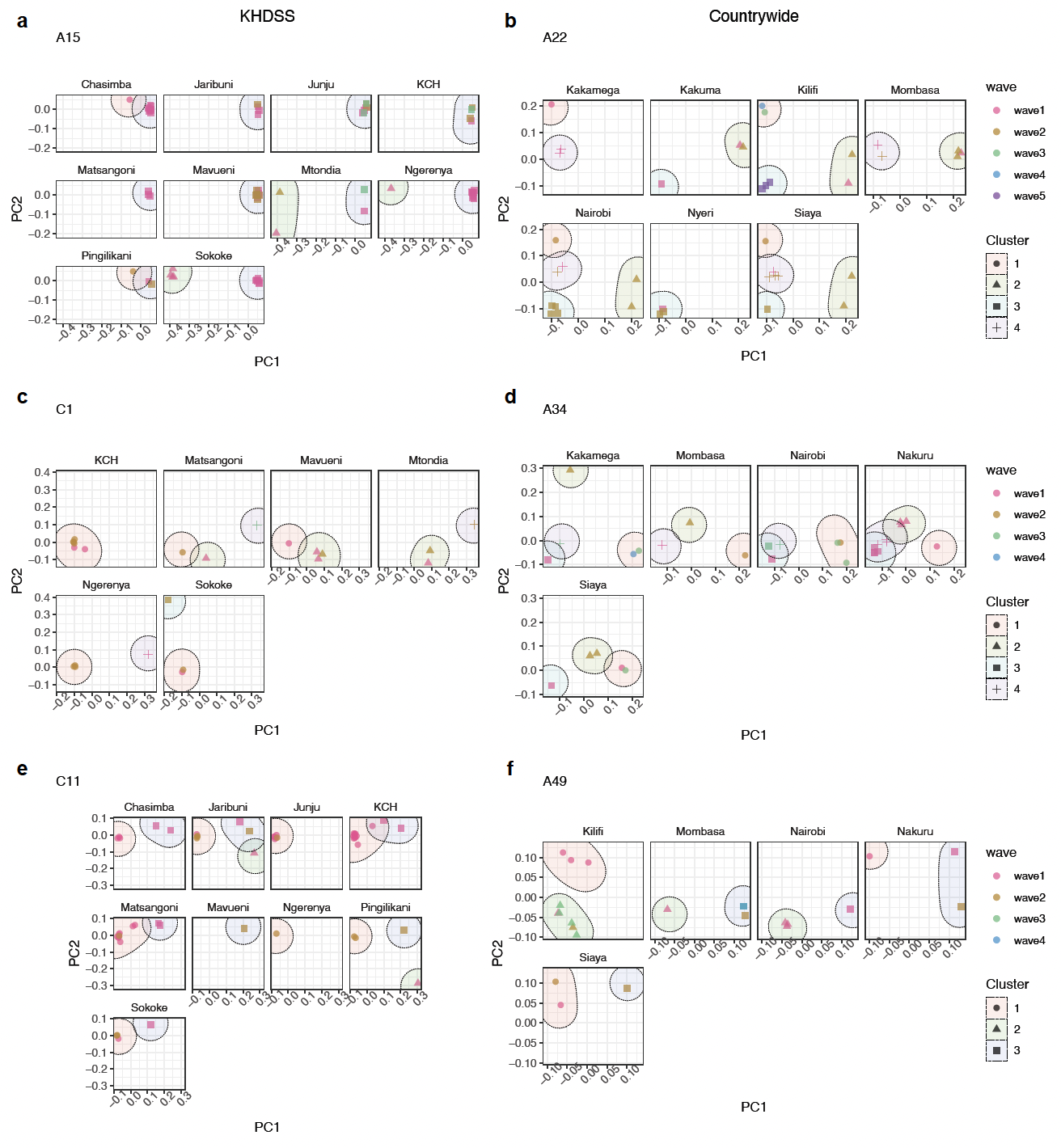


**Supplementary Figure 2.** K-means clustering of principal components (PC) performed on six select RV types stratified by RV type and administrative location. Each dot represents a sample. Samples are grouped by location (for the Kilifi HDSS study) or county (for the countrywide study) and coloured by their respective epidemic wave. The respective genetic cluster is highlighted by a shaded ellipsis.

**References**

1. Munywoki, P. K. *et al.* Continuous Invasion by Respiratory Viruses Observed in Rural Households During a Respiratory Syncytial Virus Seasonal Outbreak in Coastal Kenya. *Clinical Infectious Diseases* **67**, 1559–1567 (2018).

2. Kamau, E. *et al.* An Intensive, Active Surveillance Reveals Continuous Invasion and High Diversity  of Rhinovirus in Households. *J Infect Dis* **219**, 1049–1057 (2019).

3. Adema, I. W. *et al.* Surveillance of respiratory viruses among children attending a primary school in rural coastal Kenya. *Wellcome Open Res* **5**, (2020).

4. Luka, M. M. *et al.* Molecular epidemiology of human rhinovirus from one-year surveillance within a school setting in rural coastal Kenya. *Open Forum Infect Dis* **7**, 2020.03.09.20033019 (2020).

5. Nyiro, J. U. *et al.* Surveillance of respiratory viruses in the outpatient setting in rural coastal Kenya: baseline epidemiological observations. *Wellcome Open Res* **3**, 89 (2018).

6. Morobe, J. M. *et al.* Human rhinovirus spatial-temporal epidemiology in rural coastal Kenya, 2015-2016, observed through outpatient surveillance [version 2; peer review: 2 approved]. *Wellcome Open Res* **3**, 128 (2018).

7. Murunga, N. *et al.* Surveillance of respiratory viruses at health facilities from across Kenya, 2014. *Wellcome Open Res* **7**, 234 (2022).

8. Morobe, J. M. *et al.* Temporal distribution of rhinovirus types in multiple regions across Kenya over one-year period, 2014. *Awaiting submission* (2023).

9. Hammitt, L. L. *et al.* A preliminary study of pneumonia etiology among hospitalized children in Kenya. *Clin Infect Dis* **54 Suppl 2**, (2012).

10. Nokes, D. J. *et al.* Incidence and severity of respiratory syncytial virus pneumonia in rural Kenyan children identified through hospital surveillance. *Clin Infect Dis* **49**, 1341–1349 (2009).

11. Onyango, C. O. *et al.* Molecular epidemiology of human rhinovirus infections in Kilifi, coastal Kenya. *J Med Virol* **84**, 823–831 (2012).

12. Berkley, J. A. *et al.* Viral etiology of severe pneumonia among Kenyan infants and children. *JAMA* **303**, 2051–2057 (2010).

13. Morobe, J. M. *et al.* Trends and Intensity of Rhinovirus Invasions in Kilifi, Coastal Kenya Over a Twelve-Year Period, 2007-2018. *Open Forum Infect Dis* (2021) doi:10.1093/OFID/OFAB571.
